# Evaluation of human T-cell leukemia virus in vitro diagnostics using plasma specimens collected in Japan

**DOI:** 10.1186/s12879-023-08402-w

**Published:** 2023-06-20

**Authors:** Shigeru Kusagawa, Ai Kawana-Tachikawa, Keiji Matsubayashi, Rieko Sobata, Isao Hamaguchi

**Affiliations:** 1 AIDS Research Center, National Institute of Infectious Diseases, Tokyo, Japan; 2 Central Blood Institute, Blood Service Headquarters, Japanese Red Cross Society, Tokyo, Japan; 3 Research Center for Biological Products in the Next Generation, National Institute of Infectious Diseases, Tokyo, Japan

**Keywords:** HTLV IVDs, New generation products, Sensitivity, Specificity

## Abstract

**Background:**

In vitro diagnostics (IVDs) for primary detection test/screening of human T-cell leukemia virus (HTLV) have recently been updated to new-generation products in Japan. In this study, the performance of these products was evaluated and discussed in terms of the usability of HTLV diagnosis in Japan.

**Methods:**

The performance of 10 HTLV IVDs for primary detection test and confirmatory/discriminatory test was evaluated. Plasma specimens that had been declared ineligible for transfusion were provided by the Japanese Red Cross Blood Center.

**Results:**

The diagnostic specificity of the IVDs was 100% (160/160). Six sandwich assays resulted in all HTLV-1/HTLV-positive specimens being positive (46/46). On the other hand, one sandwich assay, IVD under development 2 (UD2), resulted in one HTLV-1-positive and one HTLV-positive specimen being negative (44/46, 95.7%). One indirect assay, HISCL HTLV-1, could not detect one HTLV-positive specimen (45/46, 97.8%), but the updated product, UD1, correctly detected it (46/46, 100%). Serodia HTLV-I, based on a particle agglutination assay, resulted in 44 of the 46 positive specimens, but could not detect two specimens (44/46, 95.7%). ESPLINE HTLV-I/II, based on an immunochromatography assay (ICA), was able to diagnose all specimens as positive (46/46, 100%).

**Conclusions:**

Six sandwich assays and an ICA demonstrated high diagnostic sensitivity and specificity and are recommended for use in HTLV diagnosis in conjunction with confirmatory/discriminatory test using the INNO-LIA HTLV-I/II Score.

**Supplementary Information:**

The online version contains supplementary material available at 10.1186/s12879-023-08402-w.

## Background

Human T-cell leukemia virus (HTLV) belongs to the family *Retroviridae*, genus *Deltaretrovirus*. Although most HTLV-1 infections are asymptomatic, some carriers develop adult T-cell leukemia (ATL) [1] and HTLV-1 associated myelopathy/tropical spastic paraparesis (HAM/TSP) [2] after a long asymptomatic period. HTLV-1 infection is endemic in southeast Japan, the Caribbean coast, South America, central Australia, and equatorial Africa [3, 4]. Based on an analysis of Japanese first-time blood donors from 2006 to 2007, approximately 1.08 million individuals were infected with HTLV-1 [5], and the estimated annual number of new HTLV-1 infections was 4,190 at the end of 2011, as reported in a retrospective cohort study of repeat blood donors who were HTLV-1-negative from 2006 to 2007 [6]. HTLV-1 mainly infects vertically through mother-to-infant transmission via breastfeeding, but can also be transmitted sexually or via blood transfusions horizontally. HTLV antibody positivity refers to an HTLV carrier and a source of HTLV infection. The anti-HTLV antibody test is useful for controlling HTLV infections.

A diagnostic test algorithm for HTLV-1 infection was established in Japan [7]. In short, primary detection test/screening of the serum or plasma obtained from peripheral blood specimens is conducted to determine a subject’s HTLV-1 infection status using commercially available HTLV-1 antibody-specific serological assays (particle agglutination (PA), chemiluminescent enzyme immunoassay (CLEIA), chemiluminescent immunoassay (CLIA), and electrochemiluminescence immunoassay (ECLIA)). Positive samples in the primary detection test were tested using a line immunoassay (LIA) as a confirmatory/discriminatory test to exclude pseudo-positive cases, because HTLV-1/2 structural protein-specific antibody reactivity can be detected by LIA. When the result is indeterminate, HTLV-1 PCR is performed to detect proviral DNA [8, 9].

Lately, HTLV in vitro diagnostics (IVDs) for primary detection tests have been updated to new-generation products based on sandwich assay. The HIV sandwich assay shows high performance because using enzymatic or chemically labeled HIV antigen instead of labeled human IgG to detect antigen–antibody complex on a carrier following bound/free separation [10, 11].

In this study, HTLV-positive reference panel, including a low signal-to-cutoff ratio (S/CO) or cutoff index (COI), and HTLV-negative reference panel were recently prepared in Japan. The performance of HTLV diagnostics for primary detection testing was evaluated using the HTLV-positive reference panel and the HTLV-negative reference panel.

## Methods

### HTLV IVDs

The performance of 10 HTLV IVDs for the primary detection test and one for confirmatory/discriminatory test was evaluated in this study. Two IVDs under development (UD1 and UD2) were included. This list is presented in Table 1.

**Table 1:** List of HTLV IVDs used in this study

IVDs	Method	Ind^g^/Sand^h^	Manufacturer	Code^i^
For primary detection test				
Architect rHTLV I/II	CLIA^a^	Sand	Abbott Japan	A
Alinity i rHTLV I/II	CLIA	Sand	Abbott Japan	B
Elecsys HTLV-I/II	ECLIA^b^	Sand	Roche Diagnostics	C
Lumipulse HTLV-I/II	CLEIA^c^	Sand	Fujirebio	D
Lumipulse Presto HTLV-I/II	CLEIA	Sand	Fujirebio	E
Serodia HTLV-I	PA^d^	-	Fujirebio	F
ESPLINE HTLV-I/II	ICA^e^	-	Fujirebio	G
HISCL HTLV-I	CLEIA	Ind	Sysmex	H
UD1	CLEIA	Sand	Sysmex	I
UD2	CLEIA	Sand	Non-disclosure	J
For confirmatory/discriminatory test				
INNO-LIA HTLV-I/II Score	LIA^f^	-	Fujirebio	

*CLIA*^a^ Chemiluminescent immunoassay, *ECLIA*^b^: electrochemiluminescence immunoassay, *CLEIA*^c^ chemiluminescent enzyme immunoassay, *PA*^d^ Particle agglutination, *ICA*^e^ Immunochromatography assay, *LIA*^f^ Line immunoassay, *Ind*^g^ Indirect assay, Sand^h^ Sandwich assay, *Code*^i^ Used in Tables 2, 3 and Additional file 1

### HTLV-positive/negative reference panels for evaluation of HTLV IVDs

To prepare HTLV-positive/negative reference panels, plasma specimens that had been declared ineligible for transfusion were provided by the Japanese Red Cross Blood Center. Additional testing after dispensing was performed according to the HTLV testing algorithm in Japan [7] at the National Institute of Infectious Diseases. The specimens were provided following an application for the use of donated blood in Japan per the guidelines on the use of donated blood in research and development. The specimen information was anonymized, and a decoding index was not created, as required by the Helsinki Declaration. Ethical approval was obtained from the Ethical Committee of the National Institute of Infectious Diseases (No. 1082).

## Results

### Estimation of the diagnostic specificity of 10 HTLV IVDs in primary detection test/screening

A total of 160 HTLV-negative plasma specimens were tested using 10 HTLV IVDs for primary detection test/screening. All IVDs were negative for all tests (Additional File 1). The diagnostic specificity of IVDs was 100%.

### Estimation of the diagnostic sensitivity of 10 HTLV IVDs in primary detection test/screening

Ten specimens, that were tested positive with relatively low S/CO or COI using multiple IVDs for primary detection test/screening and were HTLV-indeterminate or negative using INNO-LIA HTLV-I/II Score (INNO-LIA), were removed from this IVD evaluation. Forty-four HTLV-1-positive and two HTLV-positive untypable specimens (No. 16 and 26), based on INNO-LIA, were tested for evaluation (Table 2). Six sandwich assays resulted in all specimens being positive. On the other hand, UD2 resulted in one HTLV-1-positive (No. 24) and one HTLV-positive specimen (No. 16) being negative (Table 2, column J). One indirect assay, HISCL HTLV-1, could not detect one HTLV-positive specimen (No. 16), but the updated product, UD1, detected it (Table 2, columns H and I). Serodia HTLV-I detected 44 of the 46 specimens, but could not detect two specimens (No. 16 and 24, Table 2, column F). ESPLINE HTLV-I/II (ICA) was able to diagnose all positive specimens being positive (Table 2, column G). The positive agreement rates are shown in Table 3.

**Table 2:** Results of HTLV IVDs using 44 HTLV-1 positive and two HTLV-positive untypable specimens

No	IVDs for primary detection test										INNO-LIA HTLV-I/II Score							
	A	B	C	D	E	F	G	H	I	J	Confirmation				Discrimination			Result
											gag p19 I/II	gag p24 I/II	env gp46 I/II	env gp21 I/II	gag p19-I	env gp46-I	env gp46-II	
	S/CO	S/CO	COI	COI	COI	D.F.^a^	Decision	COI	COI	COI								
1	103.55	92.10	320.7	> 50.0	> 50.0	2048	Positive	> 100.0	> 100.0	239.893	3 +	2 +	2 +	2 +	2 +	3 +	±	HTLV-I
2	156.38	141.75	335.0	> 50.0	> 50.0	8192	Positive	> 100.0	> 100.0	289.531	2 +	2 +	-	3 +	2 +	3 +	±	HTLV-I
3	17.63	17.06	58.1	27.9	23.4	64	Positive	3.6	49.9	6.386	1 +	±	1 +	1 +	1 +	±	-	HTLV-I
4	46.50	43.95	178.2	> 50.0	> 50.0	512	Positive	6.8	> 100.0	6.034	1 +	-	1 +	2 +	-	1 +	-	HTLV-I
5	119.41	107.43	283.5	> 50.0	> 50.0	2048	Positive	23.8	> 100.0	55.062	2 +	1 +	3 +	2 +	1 +	±	-	HTLV-I
6	76.57	73.74	294.0	> 50.0	> 50.0	2048	Positive	85.3	> 100.0	170.378	3 +	3 +	1 +	3 +	±	3 +	±	HTLV-I
7	73.14	67.73	292.2	> 50.0	> 50.0	1024	Positive	13.8	> 100.0	33.105	1 +	1 +	1 +	2 +	±	1 +	-	HTLV-I
8	51.17	49.42	15.0	42.4	48.8	64	Positive	4.5	39.4	11.979	1 +	-	2 +	1 +	±	±	-	HTLV-I
9	54.26	51.43	130.7	> 50.0	> 50.0	256	Positive	15.5	> 100.0	39.815	2 +	1 +	2 +	2 +	2 +	±	±	HTLV-I
10	67.95	64.61	100.2	> 50.0	> 50.0	256	Positive	5.1	> 100.0	40.375	1 +	1 +	2 +	2 +	±	2 +	-	HTLV-I
11	61.10	59.17	41.9	> 50.0	> 50.0	128	Positive	12.8	> 100.0	51.587	2 +	1 +	3 +	1 +	±	2 +	-	HTLV-I
12	108.69	102.12	286.7	> 50.0	> 50.0	1024	Positive	43.3	> 100.0	114.827	2 +	1 +	1 +	3 +	2 +	3 +	-	HTLV-I
13	111.35	103.79	153.4	> 50.0	> 50.0	512	Positive	32.6	> 100.0	39.667	2 +	1 +	3 +	2 +	2 +	3 +	-	HTLV-I
14	71.64	72.32	146.3	> 50.0	> 50.0	1024	Positive	10.2	> 100.0	20.430	2 +	±	1 +	2 +	1 +	2 +	-	HTLV-I
15	117.15	110.85	311.5	> 50.0	> 50.0	4096	Positive	50.3	> 100.0	147.137	3 +	2 +	2 +	3 +	3 +	3 +	-	HTLV-I
16	2.99	2.78	1.1	3.7	1.5	0	Positive	0.0	2.2	0.205	±	-	-	±	-	-	-	HTLV
17	25.70	23.20	107.3	23.9	29.5	256	Positive	8.1	79.1	14.375	2 +	2 +	2 +	2 +	-	2 +	-	HTLV-I
18	40.27	38.15	56.7	45.2	42.6	128	Positive	9.5	74.2	9.735	1 +	-	2 +	2 +	1 +	1 +	-	HTLV-I
19	36.10	36.02	45.0	> 50.0	37.0	256	Positive	35.2	62.8	45.865	2 +	1 +	1 +	2 +	2 +	-	-	HTLV-I
20	59.80	56.40	103.3	> 50.0	> 50.0	256	Positive	12.8	> 100.0	17.582	1 +	±	2 +	2 +	1 +	1 +	-	HTLV-I
21	81.13	75.12	112.1	> 50.0	> 50.0	512	Positive	8.2	> 100.0	21.974	2 +	±	1 +	2 +	2 +	1 +	-	HTLV-I
22	12.07	11.87	56.2	21.6	22.5	128	Positive	6.5	52.2	4.381	2 +	±	2 +	2 +	-	2 +	-	HTLV-I
23	50.77	48.20	139.9	> 50.0	> 50.0	512	Positive	14.7	> 100.0	12.135	2 +	-	1 +	2 +	2 +	±	-	HTLV-I
24	3.46	3.41	7.7	2.9	1.7	0	Positive	4.7	3.5	0.542	±	-	-	1 +	1 +	-	-	HTLV-I
25	2.72	2.68	53.3	6.5	6.1	32	Positive	19.4	13.7	2.509	2 +	-	1 +	2 +	±	±	-	HTLV-I
26	58.14	57.77	65.6	> 50.0	> 50.0	256	Positive	7.7	> 100.0	16.409	2 +	-	-	2 +	-	-	-	HTLV
27	98.19	88.51	237.5	> 50.0	> 50.0	1024	Positive	26.6	> 100.0	28.569	2 +	±	2 +	2 +	2 +	2 +	-	HTLV-I
28	106.43	98.42	182.1	> 50.0	> 50.0	1024	Positive	68.4	> 100.0	107.693	2 +	2 +	2 +	2 +	2 +	3 +	±	HTLV-I
29	104.05	94.04	325.5	> 50.0	> 50.0	2048	Positive	35.0	> 100.0	61.918	2 +	1 +	2 +	3 +	-	2 +	-	HTLV-I
30	143.36	116.25	336.7	> 50.0	> 50.0	2048	Positive	> 100.0	> 100.0	242.534	2 +	2 +	2 +	2 +	2 +	3 +	1 +	HTLV-I
31	109.65	100.68	272.5	> 50.0	> 50.0	2048	Positive	6.1	> 100.0	112.804	2 +	2 +	2 +	2 +	±	1 +	-	HTLV-I
32	110.22	105.59	312.9	> 50.0	> 50.0	2048	Positive	11.8	> 100.0	47.743	2 +	2 +	±	2 +	-	1 +	-	HTLV-I
33	141.18	128.81	323.1	> 50.0	> 50.0	4096	Positive	61.2	> 100.0	160.308	2 +	2 +	2 +	3 +	1 +	3 +	±	HTLV-I
34	104.07	90.33	164.4	> 50.0	> 50.0	512	Positive	28.2	> 100.0	22.931	2 +	-	2 +	2 +	1 +	2 +	-	HTLV-I
35	142.34	129.40	304.5	> 50.0	> 50.0	8192	Positive	98.0	> 100.0	101.134	2 +	2 +	2 +	3 +	2 +	3 +	1 +	HTLV-I
36	123.75	111.83	336.8	> 50.0	> 50.0	4096	Positive	58.0	> 100.0	227.879	2 +	2 +	2 +	2 +	2 +	2 +	±	HTLV-I
37	134.56	125.49	194.8	> 50.0	> 50.0	512	Positive	11.5	> 100.0	72.161	2 +	1 +	-	2 +	1 +	-	-	HTLV-I
38	93.86	87.15	326.8	> 50.0	> 50.0	4096	Positive	77.6	> 100.0	158.547	2 +	2 +	2 +	3 +	2 +	3 +	-	HTLV-I
39	91.70	78.26	107.8	> 50.0	> 50.0	256	Positive	22.8	> 100.0	51.753	1 +	2 +	2 +	2 +	1 +	2 +	-	HTLV-I
40	128.72	121.58	355.3	> 50.0	> 50.0	2048	Positive	5.2	> 100.0	26.333	1 +	-	2 +	2 +	1 +	-	-	HTLV-I
41	103.66	92.73	80.3	> 50.0	> 50.0	256	Positive	1.8	> 100.0	38.196	2 +	1 +	1 +	2 +	1 +	-	-	HTLV-I
42	130.78	115.42	333.7	> 50.0	> 50.0	4096	Positive	69.7	> 100.0	127.553	3 +	2 +	2 +	3 +	2 +	3 +	±	HTLV-I
43	99.55	83.48	291.8	> 50.0	> 50.0	2048	Positive	72.4	> 100.0	112.354	3 +	2 +	2 +	2 +	2 +	2 +	-	HTLV-I
44	40.07	38.19	230.3	> 50.0	> 50.0	512	Positive	9.0	> 100.0	11.480	2 +	±	2 +	2 +	-	2 +	-	HTLV-I
45	63.71	61.02	106.0	> 50.0	> 50.0	512	Positive	11.1	> 100.0	15.413	2 +	±	2 +	2 +	2 +	2 +	-	HTLV-I
46	77.28	71.96	272.4	> 50.0	> 50.0	2048	Positive	83.0	> 100.0	193.647	3 +	3 +	2 +	2 +	2 +	2 +	-	HTLV-I

A: Architect rHTLV I/II; B: Alinity I rHTLV I/II; C: Elecsys HTLV-I/II; D: Lumipulse HTLV-I/II; E: Lumipulse Presto HTLV-I/II; F: Serodia HTLV-I; G: ESPLINE HTLV-I/II; H: HISCL HTLV-I; I: UD1; J: UD2

D.F.^a^: dilution factor, D.F. ≥ 16: Positive, S/CO, COI ≧ 1.0: Positive

**Table 3:** Positive agreement rate of HTLV IVDs for primary detection test/screening

	IVDs		
	A, B, C, D, E, G, I	H	F, J
HTLV(-1) positive	46	46	46
Test positive	46	45	44
Positive agreement rate (%)	100	97.8	95.7

A: Architect rHTLV I/II; B: Alinity I rHTLV I/II; C: Elecsys HTLV-I/II; D: Lumipulse HTLV-I/II; E: Lumipulse Presto HTLV-I/II; F: Serodia HTLV-I; G: ESPLINE HTLV-I/II; H: HISCL HTLV-I; I: UD1; J: UD2

### Distribution of S/CO or COI in HTLV-1 and HTLV positive specimens

**Fig. 1:**
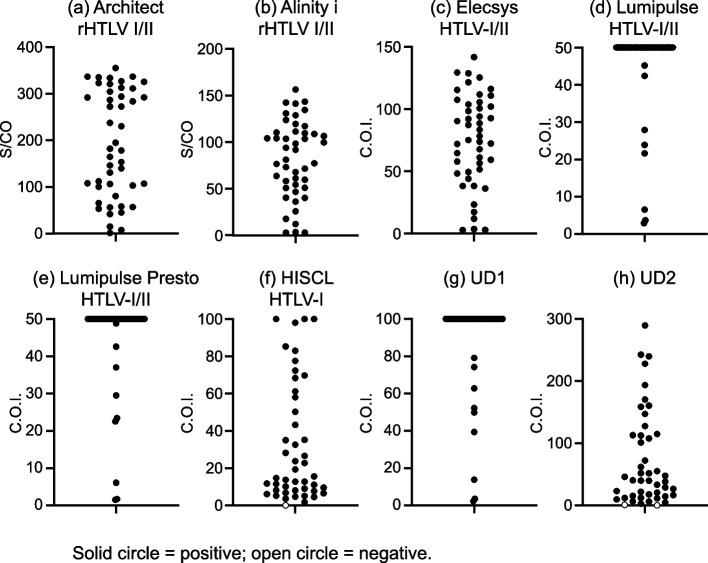
Distribution of S/CO and COI in HTLV-1 and HTLV positive specimens

The distribution of the S/CO or COI from the CLEIA, CLIA, and ECLIA tests with HTLV-1 and HTLV-positive specimens is shown in Fig. 1. The COI of UD1, based on the sandwich assay, showed a widespread distribution (Fig. 1g) compared with the approved indirect assay product, HISCL HTLV-1 (Fig. 1f). The distribution of other IVDs, based on sandwich assay, showed a similar to that of UD1 (Fig. 1a–e), except for UD2 (Fig. 1h).

## Discussion

In the present study, HTLV-positive and HTLV-negative specimen reference panels were prepared from plasma specimens that had been declared ineligible for transfusion and used for the evaluation of HTLV IVDs. The S/CO and COI of HTLV-positive specimens were widely distributed, with values around and far from the cutoff values. This specimen reference panel is useful for the evaluation of IVDs.

The diagnostic specificity of IVDs for the primary detection test/screening was examined using 160 HTLV-negative specimens. The negative agreement rate for all IVDs used in this study was 100%. These results are acceptable.

The diagnostic sensitivity of the IVDs for the primary detection test/screening was examined using 44 HTLV-1-positive and two HTLV-positive specimens, which resulted from INNO-LIA. HTLV CLEIA/CLIA/ECLIA IVDs, which were approved recently in Japan, have been updated to new-generation products based on sandwich assay. Six of the seven sandwich assays showed 100% sensitivity (46/46). HISCL HTLV-I, indirect CLEIA, could not detect one HTLV-positive specimen, and the positive agreement rate was 97.8% (45/46). However, their updated product, UD1, detected all of the specimens, and the COI was widely distributed to that of HISCL HTLV-I. The distribution of the other five sandwich assays was similar to that of UD1. The performances of the HTLV CLEIA/CLIA/ECLIA IVDs were also examined and have been highly acclaimed in previous reports [12–18]. These results indicated that the update to sandwich assay was effective for the improvement of HTLV IVD performance, similar to HIV IVDs [10, 11], and the updated products were recommended for primary detection test/screening of HTLV infection.

Specimens no.16 and 24 showed negative results using some IVDs for primary detection test/screening. No.16 showed p19 I/II ( ±), gp21 I/II ( ±), and no.24 was p19 I/II ( ±), gp21 I/II (1 +), p19 I (1 +) using INNO-LIA. HISCL HTLV-I, that decided specimen no.16 to be negative, use HTLV-1 p19 and gp46 for detection of anti-HTLV-1 antibodies. Serodia HTLV-I use purified HTLV-1 viral antigen and UD2 use HTLV-1/2 gp21 and p24. Those IVDs tested negative both no.16 and 24. These results suggested that the kind of the antigen used in these kits is not necessarily related to the contradiction with the test result of INNO-LIA.

Eight of the ten HTLV IVDs for primary detection test/screening used in this study are based on chemiluminescence detection and require a specific analyzer. These IVDs have the following advantages: (1) The process after setting the specimens is performed automatically, preventing human error and enabling tests to be performed with consistent quality. (2) More specimens can be tested with fewer personnel. (3) Use of the IVDs does not require special manual skills, reducing the burden of education and training. (4) They are compatible with the automation of examination reception and result output. The sensitivity and specificity of IVDs are high, particularly in sandwich assays; thus, the performance is considered to be adequate. Because a system for transporting clinical specimens is in place in Japan, consolidating laboratory operations in large-scale medical facilities and testing laboratories facilitates more efficient processing of multiple specimens.

By contrast, using these IVDs in small- and medium-sized laboratories and areas with low HTLV prevalence is difficult because they are expensive to purchase and maintain. ESPLINE HTLV-I/II is based on ICA and may be suitable for HTLV primary detection test/screening in small- and medium-sized diagnostic laboratories. Its performance was superior to that of Serodia HTLV-I, the only manual-use IVD approved in Japan. Its performance was equivalent to the new-generation CLEIA/CLIA/ECLIA tests in this study. Incorporating ESPLINE HTLV-I/II into the HTLV testing algorithm will be a subject in the future.

INNO-LIA can detect HTLV-1/2 structural protein-specific antibody responses and is suitable for confirmatory/discriminatory test. In previous studies, INNO-LIA has been reported to display superior sensitivity and specificity compared to the previously used WB-based diagnostics [7, 19–21] and has been incorporated into the recommended testing algorithm in Japan [7]. In the present study, 10 specimens tested positive for relatively low S/CO or COI in multiple IVDs in primary detection test/screening and were HTLV-indeterminate or negative upon INNO-LIA testing. Primary detection test/screening positive and INNO-LIA-indeterminate or negative cases have also been reported [7, 17–21]. Because these were plasma specimens, proviral testing could not be performed, and these specimens could not be identified as HTLV-positive. Therefore, we believe it is reasonable to exclude these specimens from IVD evaluation for primary detection test/screening.

We have included an entry of IVDs under development in this project and provided its evaluation results. We believe this type of research project contributes to the performance confirmation of approved IVDs and to improving the performance of new IVDs. We are still collecting new specimens. We will plan to continue evaluating approved HTLV IVDs periodically.

## Conclusions

CLEIA/CLIA/ECLIA IVDs allow for automatic and rapid multi-specimen results without complex manipulation. New-generation HTLV IVDs, based on sandwich assay, demonstrated HTLV testing capabilities with high sensitivity and specificity. They are recommended for use in HTLV primary detection test/screening. However, these IVDs are expensive to purchase and maintain for small- and medium-sized laboratories. The use of ICA, which showed as same high performance as these IVDs in this study may overcome this issue.

## Supplementary Information

**Additional file 1.** Results of HTLV IVDs for primary detection test using HTLV-negative reference panel.

## Data Availability

The dataset used in this study is available from the corresponding author upon request.

## Abbreviations

HTLV: Human T-cell leukemia virus
IVD: In vitro diagnostics
S/CO: Signal to cutoff ratio
COI: Cutoff index
ATL: Adult T-cell leukemia
HAM: HTLV-1 associated myelopathy
TSP: Tropical spastic paraparesis
PA: Particle agglutination
ICA: Immunochromatography assay
CLEIA: Chemiluminescent enzyme immunoassay
CLIA: Chemiluminescent immunoassay
ECLIA: Electrochemiluminescence immunoassay
LIA: Line immunoassay
INNO-LIA: INNO-LIA HTLV-I/II Score
UD: IVD under development
