# Supplementary material for: Evaluation of human T-cell leukemia virus in vitro diagnostics using plasma specimens collected in Japan
Source: BMC Infect Dis. 2023 Jun 20;23:418. doi: 10.1186/s12879-023-08402-w (PMC10283183; doi:10.1186/s12879-023-08402-w)
Supplement: Supplementary file 1 — Additional file 1. Results of HTLV IVDs for primary detection test using HTLV-negative reference panel. [file 12879_2023_8402_MOESM1_ESM.docx]

Additional file 1. Results of HTLV IVDs for primary detection test using HTLV-negative reference panel

| No. | IVDs for primary detection test | | | | | | | | | |
| --- | --- | --- | --- | --- | --- | --- | --- | --- | --- | --- |
|  | A | B | C | D | E | F | G | H | I | J |
|  | S/CO | S/CO | C.O.I. | C.O.I. | C.O.I. | D.F.† | Decision | C.O.I. | C.O.I. | C.O.I. |
| 1 | 0.085 | 0.105 | 0.089 | 0.1 | 0.2 | - | Negative | 0.0 | 0.0 | 0.009 |
| 2 | 0.100 | 0.100 | 0.097 | 0.1 | 0.3 | - | Negative | 0.0 | 0.0 | 0.008 |
| 3 | 0.095 | 0.090 | 0.093 | 0.1 | 0.3 | - | Negative | 0.0 | 0.0 | 0.010 |
| 4 | 0.085 | 0.085 | 0.092 | 0.1 | 0.3 | - | Negative | 0.1 | 0.0 | 0.009 |
| 5 | 0.090 | 0.105 | 0.882 | 0.1 | 0.4 | - | Negative | 0.0 | 0.2 | 0.014 |
| 6 | 0.110 | 0.100 | 0.092 | 0.1 | 0.3 | - | Negative | 0.0 | 0.0 | 0.011 |
| 7 | 0.125 | 0.100 | 0.094 | 0.1 | 0.2 | - | Negative | 0.0 | 0.0 | 0.014 |
| 8 | 0.075 | 0.080 | 0.096 | 0.1 | 0.2 | - | Negative | 0.6 | 0.0 | 0.008 |
| 9 | 0.105 | 0.095 | 0.089 | 0.1 | 0.5 | - | Negative | 0.0 | 0.0 | 0.011 |
| 10 | 0.140 | 0.105 | 0.093 | 0.1 | 0.3 | - | Negative | 0.0 | 0.0 | 0.008 |
| 11 | 0.075 | 0.090 | 0.092 | 0.1 | 0.1 | - | Negative | 0.0 | 0.0 | 0.014 |
| 12 | 0.080 | 0.085 | 0.089 | 0.1 | 0.1 | - | Negative | 0.0 | 0.0 | 0.014 |
| 13 | 0.090 | 0.090 | 0.270 | 0.1 | 0.3 | - | Negative | 0.0 | 0.0 | 0.012 |
| 14 | 0.115 | 0.085 | 0.092 | 0.1 | 0.2 | - | Negative | 0.0 | 0.0 | 0.009 |
| 15 | 0.080 | 0.085 | 0.090 | 0.1 | 0.1 | - | Negative | 0.0 | 0.0 | 0.013 |
| 16 | 0.100 | 0.095 | 0.089 | 0.1 | 0.3 | - | Negative | 0.0 | 0.0 | 0.011 |
| 17 | 0.090 | 0.095 | 0.095 | 0.1 | 0.2 | - | Negative | 0.0 | 0.0 | 0.010 |
| 18 | 0.110 | 0.095 | 0.091 | 0.1 | 0.2 | - | Negative | 0.0 | 0.0 | 0.011 |
| 19 | 0.085 | 0.100 | 0.103 | 0.1 | 0.2 | - | Negative | 0.0 | 0.0 | 0.010 |
| 20 | 0.075 | 0.085 | 0.091 | 0.1 | 0.5 | - | Negative | 0.0 | 0.0 | 0.008 |
| 21 | 0.100 | 0.085 | 0.089 | 0.1 | 0.4 | - | Negative | 0.0 | 0.0 | 0.009 |
| 22 | 0.145 | 0.125 | 0.089 | 0.1 | 0.3 | - | Negative | 0.0 | 0.0 | 0.010 |
| 23 | 0.070 | 0.080 | 0.094 | 0.1 | 0.2 | - | Negative | 0.0 | 0.0 | 0.018 |
| 24 | 0.255 | 0.255 | 0.111 | 0.2 | 0.5 | - | Negative | 0.9 | 0.1 | 0.009 |
| 25 | 0.090 | 0.090 | 0.088 | 0.1 | 0.3 | - | Negative | 0.0 | 0.0 | 0.012 |
| 26 | 0.075 | 0.095 | 0.089 | 0.1 | 0.2 | - | Negative | 0.0 | 0.0 | 0.013 |
| 27 | 0.105 | 0.105 | 0.090 | 0.1 | 0.4 | - | Negative | 0.0 | 0.0 | 0.008 |
| 28 | 0.075 | 0.090 | 0.091 | 0.1 | 0.4 | - | Negative | 0.0 | 0.0 | 0.014 |
| 29 | 0.085 | 0.090 | 0.091 | 0.1 | 0.2 | - | Negative | 0.0 | 0.0 | 0.012 |
| 30 | 0.120 | 0.115 | 0.091 | 0.1 | 0.2 | - | Negative | 0.0 | 0.0 | 0.008 |
| 31 | 0.075 | 0.090 | 0.092 | 0.1 | 0.4 | - | Negative | 0.0 | 0.0 | 0.009 |
| 32 | 0.120 | 0.085 | 0.099 | 0.1 | 0.2 | - | Negative | 0.0 | 0.0 | 0.013 |
| 33 | 0.115 | 0.125 | 0.097 | 0.1 | 0.2 | - | Negative | 0.1 | 0.0 | 0.020 |
| 34 | 0.075 | 0.095 | 0.094 | 0.1 | 0.3 | - | Negative | 0.0 | 0.0 | 0.008 |
| 35 | 0.090 | 0.095 | 0.094 | 0.1 | 0.4 | - | Negative | 0.0 | 0.0 | 0.010 |
| 36 | 0.075 | 0.085 | 0.090 | 0.1 | 0.2 | - | Negative | 0.0 | 0.0 | 0.010 |
| 37 | 0.070 | 0.095 | 0.090 | 0.1 | 0.2 | - | Negative | 0.1 | 0.0 | 0.010 |
| 38 | 0.110 | 0.095 | 0.090 | 0.1 | 0.2 | - | Negative | 0.0 | 0.0 | 0.011 |
| 39 | 0.090 | 0.100 | 0.102 | 0.1 | 0.2 | - | Negative | 0.0 | 0.0 | 0.009 |
| 40 | 0.100 | 0.085 | 0.091 | 0.1 | 0.1 | - | Negative | 0.0 | 0.0 | 0.010 |
| 41 | 0.110 | 0.100 | 0.092 | 0.1 | 0.6 | - | Negative | 0.0 | 0.0 | 0.013 |
| 42 | 0.060 | 0.095 | 0.093 | 0.1 | 0.2 | - | Negative | 0.0 | 0.0 | 0.017 |
| 43 | 0.120 | 0.110 | 0.091 | 0.1 | 0.2 | - | Negative | 0.0 | 0.0 | 0.013 |
| 44 | 0.115 | 0.085 | 0.090 | 0.1 | 0.3 | - | Negative | 0.0 | 0.0 | 0.009 |
| 45 | 0.080 | 0.100 | 0.090 | 0.1 | 0.2 | - | Negative | 0.0 | 0.0 | 0.009 |
| 46 | 0.090 | 0.090 | 0.092 | 0.1 | 0.2 | - | Negative | 0.0 | 0.0 | 0.012 |
| 47 | 0.075 | 0.100 | 0.093 | 0.1 | 0.2 | - | Negative | 0.0 | 0.0 | 0.008 |
| 48 | 0.080 | 0.095 | 0.092 | 0.1 | 0.2 | - | Negative | 0.0 | 0.0 | 0.010 |
| 49 | 0.075 | 0.090 | 0.090 | 0.1 | 0.1 | - | Negative | 0.0 | 0.0 | 0.017 |
| 50 | 0.170 | 0.125 | 0.114 | 0.1 | 0.3 | - | Negative | 0.0 | 0.0 | 0.012 |
| 51 | 0.115 | 0.115 | 0.091 | 0.1 | 0.5 | - | Negative | 0.0 | 0.1 | 0.008 |
| 52 | 0.070 | 0.100 | 0.093 | 0.1 | 0.3 | - | Negative | 0.0 | 0.1 | 0.009 |
| 53 | 0.115 | 0.115 | 0.091 | 0.1 | 0.1 | - | Negative | 0.1 | 0.1 | 0.012 |
| 54 | 0.115 | 0.110 | 0.091 | 0.1 | 0.2 | - | Negative | 0.0 | 0.1 | 0.010 |
| 55 | 0.070 | 0.095 | 0.091 | 0.1 | 0.1 | - | Negative | 0.0 | 0.1 | 0.009 |
| 56 | 0.075 | 0.095 | 0.089 | 0.1 | 0.1 | - | Negative | 0.0 | 0.1 | 0.010 |
| 57 | 0.090 | 0.095 | 0.089 | 0.1 | 0.1 | - | Negative | 0.0 | 0.1 | 0.008 |
| 58 | 0.095 | 0.095 | 0.091 | 0.1 | 0.1 | - | Negative | 0.0 | 0.1 | 0.010 |
| 59 | 0.100 | 0.080 | 0.092 | 0.1 | 0.2 | - | Negative | 0.0 | 0.1 | 0.010 |
| 60 | 0.085 | 0.105 | 0.091 | 0.1 | 0.3 | - | Negative | 0.0 | 0.1 | 0.015 |
| 61 | 0.095 | 0.105 | 0.091 | 0.1 | 0.3 | - | Negative | 0.0 | 0.1 | 0.008 |
| 62 | 0.135 | 0.110 | 0.092 | 0.1 | 0.3 | - | Negative | 0.0 | 0.1 | 0.018 |
| 63 | 0.115 | 0.115 | 0.094 | 0.1 | 0.3 | - | Negative | 0.9 | 0.1 | 0.008 |
| 64 | 0.080 | 0.095 | 0.094 | 0.1 | 0.2 | - | Negative | 0.0 | 0.1 | 0.015 |
| 65 | 0.100 | 0.110 | 0.095 | 0.1 | 0.2 | - | Negative | 0.0 | 0.1 | 0.017 |
| 66 | 0.125 | 0.125 | 0.095 | 0.1 | 0.3 | - | Negative | 0.0 | 0.1 | 0.008 |
| 67 | 0.085 | 0.090 | 0.094 | 0.1 | 0.3 | - | Negative | 0.0 | 0.1 | 0.011 |
| 68 | 0.065 | 0.105 | 0.099 | 0.1 | 0.3 | - | Negative | 0.0 | 0.1 | 0.009 |
| 69 | 0.065 | 0.090 | 0.094 | 0.1 | 0.3 | - | Negative | 0.0 | 0.1 | 0.008 |
| 70 | 0.095 | 0.095 | 0.096 | 0.1 | 0.2 | - | Negative | 0.0 | 0.1 | 0.008 |
| 71 | 0.095 | 0.115 | 0.092 | 0.1 | 0.3 | - | Negative | 0.0 | 0.1 | 0.015 |
| 72 | 0.160 | 0.165 | 0.093 | 0.1 | 0.4 | - | Negative | 0.0 | 0.1 | 0.014 |
| 73 | 0.150 | 0.140 | 0.096 | 0.1 | 0.5 | - | Negative | 0.0 | 0.1 | 0.010 |
| 74 | 0.085 | 0.090 | 0.095 | 0.1 | 0.5 | - | Negative | 0.0 | 0.1 | 0.008 |
| 75 | 0.065 | 0.100 | 0.094 | 0.1 | 0.3 | - | Negative | 0.6 | 0.1 | 0.008 |
| 76 | 0.110 | 0.120 | 0.092 | 0.1 | 0.3 | - | Negative | 0.0 | 0.1 | 0.010 |
| 77 | 0.090 | 0.100 | 0.094 | 0.1 | 0.2 | - | Negative | 0.1 | 0.1 | 0.014 |
| 78 | 0.085 | 0.095 | 0.091 | 0.1 | 0.4 | - | Negative | 0.0 | 0.1 | 0.011 |
| 79 | 0.090 | 0.100 | 0.094 | 0.1 | 0.2 | - | Negative | 0.0 | 0.1 | 0.008 |
| 80 | 0.090 | 0.100 | 0.093 | 0.1 | 0.4 | - | Negative | 0.0 | 0.1 | 0.012 |
| 81 | 0.090 | 0.085 | 0.092 | 0.1 | 0.2 | - | Negative | 0.0 | 0.0 | 0.009 |
| 82 | 0.110 | 0.095 | 0.090 | 0.1 | 0.4 | - | Negative | 0.0 | 0.0 | 0.008 |
| 83 | 0.105 | 0.090 | 0.091 | 0.1 | 0.4 | - | Negative | 0.0 | 0.0 | 0.010 |
| 84 | 0.085 | 0.110 | 0.089 | 0.1 | 0.4 | - | Negative | 0.0 | 0.0 | 0.009 |
| 85 | 0.130 | 0.115 | 0.093 | 0.1 | 0.4 | - | Negative | 0.0 | 0.0 | 0.011 |
| 86 | 0.090 | 0.090 | 0.091 | 0.1 | 0.5 | - | Negative | 0.0 | 0.0 | 0.009 |
| 87 | 0.090 | 0.095 | 0.093 | 0.1 | 0.3 | - | Negative | 0.0 | 0.0 | 0.011 |
| 88 | 0.130 | 0.120 | 0.091 | 0.1 | 0.2 | - | Negative | 0.0 | 0.0 | 0.009 |
| 89 | 0.115 | 0.115 | 0.092 | 0.1 | 0.4 | - | Negative | 0.0 | 0.0 | 0.008 |
| 90 | 0.140 | 0.115 | 0.090 | 0.1 | 0.5 | - | Negative | 0.0 | 0.0 | 0.014 |
| 91 | 0.115 | 0.095 | 0.092 | 0.1 | 0.3 | - | Negative | 0.0 | 0.0 | 0.009 |
| 92 | 0.080 | 0.090 | 0.092 | 0.1 | 0.3 | - | Negative | 0.0 | 0.0 | 0.015 |
| 93 | 0.080 | 0.095 | 0.150 | 0.1 | 0.5 | - | Negative | 0.0 | 0.0 | 0.015 |
| 94 | 0.115 | 0.090 | 0.089 | 0.1 | 0.3 | - | Negative | 0.0 | 0.0 | 0.008 |
| 95 | 0.100 | 0.085 | 0.091 | 0.1 | 0.6 | - | Negative | 0.0 | 0.0 | 0.014 |
| 96 | 0.065 | 0.080 | 0.093 | 0.1 | 0.2 | - | Negative | 0.0 | 0.0 | 0.008 |
| 97 | 0.090 | 0.105 | 0.092 | 0.1 | 0.4 | - | Negative | 0.0 | 0.0 | 0.018 |
| 98 | 0.105 | 0.105 | 0.092 | 0.1 | 0.2 | - | Negative | 0.0 | 0.0 | 0.011 |
| 99 | 0.095 | 0.095 | 0.093 | 0.1 | 0.2 | - | Negative | 0.0 | 0.0 | 0.011 |
| 100 | 0.065 | 0.090 | 0.089 | 0.1 | 0.3 | - | Negative | 0.0 | 0.0 | 0.008 |
| 101 | 0.075 | 0.080 | 0.091 | 0.1 | 0.6 | - | Negative | 0.0 | 0.0 | 0.011 |
| 102 | 0.105 | 0.105 | 0.090 | 0.1 | 0.4 | - | Negative | 0.0 | 0.0 | 0.012 |
| 103 | 0.100 | 0.110 | 0.093 | 0.1 | 0.4 | - | Negative | 0.0 | 0.2 | 0.017 |
| 104 | 0.090 | 0.090 | 0.091 | 0.1 | 0.4 | - | Negative | 0.0 | 0.0 | 0.013 |
| 105 | 0.075 | 0.095 | 0.092 | 0.1 | 0.2 | - | Negative | 0.0 | 0.0 | 0.013 |
| 106 | 0.075 | 0.090 | 0.090 | 0.1 | 0.2 | - | Negative | 0.0 | 0.1 | 0.009 |
| 107 | 0.100 | 0.100 | 0.091 | 0.1 | 0.6 | - | Negative | 0.0 | 0.1 | 0.008 |
| 108 | 0.090 | 0.095 | 0.093 | 0.1 | 0.2 | - | Negative | 0.0 | 0.0 | 0.007 |
| 109 | 0.080 | 0.090 | 0.090 | 0.1 | 0.2 | - | Negative | 0.0 | 0.0 | 0.019 |
| 110 | 0.110 | 0.105 | 0.091 | 0.1 | 0.3 | - | Negative | 0.0 | 0.0 | 0.008 |
| 111 | 0.115 | 0.090 | 0.128 | 0.1 | 0.2 | - | Negative | 0.0 | 0.0 | 0.008 |
| 112 | 0.090 | 0.085 | 0.093 | 0.1 | 0.5 | - | Negative | 0.0 | 0.0 | 0.014 |
| 113 | 0.105 | 0.100 | 0.094 | 0.1 | 0.2 | - | Negative | 0.0 | 0.0 | 0.014 |
| 114 | 0.100 | 0.100 | 0.091 | 0.1 | 0.5 | - | Negative | 0.0 | 0.0 | 0.010 |
| 115 | 0.090 | 0.090 | 0.093 | 0.1 | 0.4 | - | Negative | 0.0 | 0.0 | 0.009 |
| 116 | 0.595 | 0.550 | 0.356 | 0.1 | 0.3 | - | Negative | 0.0 | 0.1 | 0.010 |
| 117 | 0.085 | 0.090 | 0.092 | 0.1 | 0.3 | - | Negative | 0.0 | 0.0 | 0.011 |
| 118 | 0.095 | 0.090 | 0.093 | 0.1 | 0.4 | - | Negative | 0.0 | 0.0 | 0.008 |
| 119 | 0.085 | 0.080 | 0.092 | 0.1 | 0.2 | - | Negative | 0.0 | 0.0 | 0.010 |
| 120 | 0.095 | 0.105 | 0.090 | 0.1 | 0.4 | - | Negative | 0.0 | 0.0 | 0.009 |
| 121 | 0.095 | 0.090 | 0.091 | 0.1 | 0.1 | - | Negative | 0.0 | 0.0 | 0.010 |
| 122 | 0.120 | 0.110 | 0.090 | 0.1 | 0.3 | - | Negative | 0.0 | 0.0 | 0.008 |
| 123 | 0.100 | 0.110 | 0.092 | 0.1 | 0.2 | - | Negative | 0.0 | 0.0 | 0.011 |
| 124 | 0.080 | 0.095 | 0.092 | 0.1 | 0.7 | - | Negative | 0.0 | 0.0 | 0.013 |
| 125 | 0.085 | 0.090 | 0.091 | 0.1 | 0.2 | - | Negative | 0.0 | 0.0 | 0.009 |
| 126 | 0.130 | 0.105 | 0.090 | 0.1 | 0.3 | - | Negative | 0.0 | 0.0 | 0.015 |
| 127 | 0.070 | 0.085 | 0.092 | 0.1 | 0.4 | - | Negative | 0.0 | 0.0 | 0.010 |
| 128 | 0.090 | 0.095 | 0.093 | 0.1 | 0.5 | - | Negative | 0.0 | 0.1 | 0.016 |
| 129 | 0.125 | 0.105 | 0.090 | 0.1 | 0.2 | - | Negative | 0.0 | 0.0 | 0.019 |
| 130 | 0.090 | 0.090 | 0.092 | 0.1 | 0.3 | - | Negative | 0.0 | 0.0 | 0.008 |
| 131 | 0.090 | 0.095 | 0.090 | 0.1 | 0.2 | - | Negative | 0.0 | 0.1 | 0.013 |
| 132 | 0.075 | 0.090 | 0.092 | 0.1 | 0.3 | - | Negative | 0.0 | 0.1 | 0.013 |
| 133 | 0.115 | 0.095 | 0.093 | 0.1 | 0.3 | - | Negative | 0.0 | 0.1 | 0.013 |
| 134 | 0.100 | 0.100 | 0.091 | 0.1 | 0.2 | - | Negative | 0.0 | 0.1 | 0.022 |
| 135 | 0.085 | 0.095 | 0.091 | 0.1 | 0.3 | - | Negative | 0.0 | 0.1 | 0.008 |
| 136 | 0.085 | 0.100 | 0.102 | 0.1 | 0.2 | - | Negative | 0.0 | 0.2 | 0.013 |
| 137 | 0.110 | 0.100 | 0.092 | 0.1 | 0.1 | - | Negative | 0.0 | 0.1 | 0.017 |
| 138 | 0.080 | 0.085 | 0.092 | 0.1 | 0.5 | - | Negative | 0.0 | 0.1 | 0.008 |
| 139 | 0.065 | 0.090 | 0.094 | 0.1 | 0.5 | - | Negative | 0.0 | 0.1 | 0.016 |
| 140 | 0.150 | 0.155 | 0.103 | 0.1 | 0.4 | - | Negative | 0.0 | 0.2 | 0.009 |
| 141 | 0.095 | 0.090 | 0.092 | 0.1 | 0.3 | - | Negative | 0.0 | 0.1 | 0.012 |
| 142 | 0.100 | 0.100 | 0.133 | 0.1 | 0.5 | - | Negative | 0.0 | 0.1 | 0.023 |
| 143 | 0.070 | 0.100 | 0.093 | 0.1 | 0.3 | - | Negative | 0.0 | 0.1 | 0.008 |
| 144 | 0.130 | 0.105 | 0.094 | 0.1 | 0.2 | - | Negative | 0.0 | 0.1 | 0.008 |
| 145 | 0.070 | 0.090 | 0.092 | 0.1 | 0.5 | - | Negative | 0.0 | 0.1 | 0.025 |
| 146 | 0.150 | 0.145 | 0.097 | 0.1 | 0.2 | - | Negative | 0.1 | 0.1 | 0.013 |
| 147 | 0.080 | 0.090 | 0.093 | 0.1 | 0.6 | - | Negative | 0.0 | 0.1 | 0.008 |
| 148 | 0.105 | 0.105 | 0.095 | 0.1 | 0.1 | - | Negative | 0.0 | 0.1 | 0.009 |
| 149 | 0.090 | 0.095 | 0.092 | 0.1 | 0.2 | - | Negative | 0.0 | 0.1 | 0.009 |
| 150 | 0.110 | 0.110 | 0.093 | 0.1 | 0.2 | - | Negative | 0.0 | 0.1 | 0.008 |
| 151 | 0.145 | 0.115 | 0.106 | 0.1 | 0.4 | - | Negative | 0.0 | 0.1 | 0.008 |
| 152 | 0.110 | 0.100 | 0.093 | 0.1 | 0.1 | - | Negative | 0.0 | 0.1 | 0.016 |
| 153 | 0.105 | 0.090 | 0.094 | 0.1 | 0.2 | - | Negative | 0.0 | 0.1 | 0.010 |
| 154 | 0.080 | 0.105 | 0.091 | 0.1 | 0.2 | - | Negative | 0.0 | 0.1 | 0.013 |
| 155 | 0.090 | 0.095 | 0.092 | 0.1 | 0.4 | - | Negative | 0.0 | 0.1 | 0.008 |
| 156 | 0.080 | 0.090 | 0.093 | 0.1 | 0.3 | - | Negative | 0.2 | 0.1 | 0.008 |
| 157 | 0.090 | 0.090 | 0.092 | 0.1 | 0.2 | - | Negative | 0.0 | 0.1 | 0.012 |
| 158 | 0.140 | 0.115 | 0.097 | 0.1 | 0.3 | - | Negative | 0.0 | 0.1 | 0.012 |
| 159 | 0.095 | 0.100 | 0.091 | 0.1 | 0.2 | - | Negative | 0.0 | 0.1 | 0.008 |
| 160 | 0.130 | 0.095 | 0.093 | 0.1 | 0.2 | - | Negative | 0.0 | 0.1 | 0.011 |

A: Architect rHTLV I/II, B: Alinity I rHTLV I/II, C: Elecsys HTLV-I/II, D: Lumipulse HTLV-I/II, E: Lumipulse Presto HTLV-I/II, F: Serodia HTLV-I, G: ESPLINE HTLV-I/II, H: HISCL HTLV-I, I: UD1, J: UD2

D.F.†: Dilution factor, D.F. ≥ 16: Positive

S/CO, C.O.I. ≧ 1.0: Positive
